# The Management of Unresectable Intrathecal Catheter-Tip-Associated Granuloma Using Morphine Therapy Cessation and Spinal Cord Stimulation

**DOI:** 10.7759/cureus.10160

**Published:** 2020-08-31

**Authors:** Maximilian Bschorer, Mauricio Martinez-Moreno, Marc Tietke, Oliver Heese

**Affiliations:** 1 Neurosurgery, Helios Kliniken Schwerin, Schwerin, DEU; 2 Neuroradiology, Helios Kliniken Schwerin, Schwerin, DEU

**Keywords:** catheter-tip inflammatory mass, intrathecal granuloma, complication, intrathecal morphine therapy, spinal cord stimulation

## Abstract

Catheter-tip-associated granulomas (CTG) are a rare but serious complication of intrathecal analgesic delivery pumps (IADP), which interfere with pain modulation and can cause irreversible neurologic deficits. The treatment of symptomatic CTG generally involves surgical resection and catheter removal. We present a case of an unresectable CTG, which we managed using progressive lowering of the intrathecal morphine sulfate (ITMS) dosage as well as spinal cord stimulation (SCS). A 55-year-old female with failed back surgery syndrome (FBSS) presented with new-onset left-sided lumbar radiculopathy after five years of moderately successful ITMS therapy. An MRI study suggested an unknown mass associated with the tip of the catheter. The tumor’s adherence to nerve roots of the conus terminalis prevented a complete resection and only allowed for a biopsy. After the SCS implantation, we progressively lowered the ITMS dose. In a follow-up consultation, the patient reported the regression of the radiculopathy as well as satisfactory pain levels without oral opiates. In this case of CTG, cessation of intrathecal morphine prevented the further growth of the granuloma. SCS effectively addressed both the chronic lumbar back pain as well as the new-onset radicular pain caused by the CTG.

## Introduction

Intrathecal analgesic delivery pumps (IADP) allow for the direct stimulation of spinal opioid receptors. Apart from allowing for greatly reduced systemic opioid doses, this method of drug administration also provides a constant drug delivery over the course of weeks and months. However, complications can arise from surgical implantation of the pump, including mechanical problems as well as pharmacological side-effects [1]. An inflammatory response at the tip of the catheter can evolve into a catheter-tip-associated granuloma (CTG), possibly leading to irreversible neurological damage [2]. Patients with CTG commonly present with worsened lower back pain, new-onset leg pain, or progressive paraplegia [3]. Catheter revision and relocation, reducing the intrathecal morphine sulfate (ITMS) concentration, or replacing the current opioid with a different opioid or non-opioid drugs are the treatment options generally available for CTG [4]. We present a case of a patient with an unresectable symptomatic CTG. In this case, the exacerbated chronic lumbar pain and the new radicular pain both responded well to spinal cord stimulation (SCS), which allowed for the cessation of intrathecal analgesic therapy.

## Case presentation

**Figure 1:**
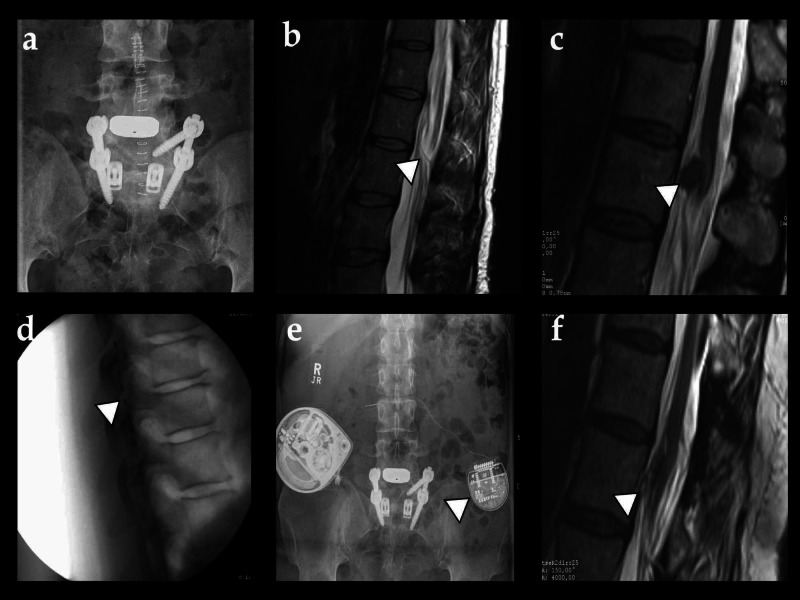
Image series a) Lumbar PA X-ray of the patient after undergoing lumbar fusion L4-L5 and, later on, extended fusion L4-S1. b) T2-weighted MRI scan of the thoracolumbar transmission in the sagittal view showing the catheter for intrathecal morphine therapy within the spinal canal (arrowhead). c) After the patient-reported new-onset left-sided radicular pain, an MRI scan revealed a tumorous formation associated with the catheter tip and the conus terminalis (arrowhead). d) Lateral view image of the intraoperative fluoroscopy of the SCS epidural electrode implanted in the patient. e) Abdominal PA X-ray showing the implanted neurostimulator (device on the left, arrowhead) and the IADP (device on the right). f) A late T2-weighted MRI scan revealed a significant reduction of the granuloma size after the progressive reduction of the morphine dose in the pump and SCS therapy PA: posteroanterior; MRI: magnetic resonance imaging; SCS: spinal cord stimulation; IADP: intrathecal analgesic delivery pump

A 55-year-old female with chronic back pain due to failed back surgery syndrome (FBSS) and a history of oral opiate dependency presented with a CTG. The patient initially underwent combined ventral and dorsal spinal fusion of the L4 and L5 vertebrae in 2005 after a decade of persistent lower back pain due to extensive osteochondrosis. Five years later, the patient received surgical treatment for a herniated spinal disc at levels L5-S1. Due to adjacent segment degeneration, the patient underwent L5-S1 spinal fusion two years later (Figure 1a).

In 2013, the patient underwent implantation of an IADP and received ITMS with satisfactory pain relief (Figure 1b). Despite an initially positive response to ITMS therapy, progressively higher doses of morphine sulfate were required, up to a daily dose of 4.8 mg with a concentration of 20 mg/ml. In 2017, the patient reported worsening lumbar pain as well as new-onset left-sided radicular pain. An MRI scan revealed a homogenous contrast agent enhancement of soft tissue in close relation with the tip of the catheter (Figure 1c). The findings suggested an inflammatory granulomatous mass. The initial treatment involved the reduction of the morphine sulfate concentration within the pump to 10 mg/ml. Unfortunately, the radicular pain continued to worsen, and the patient developed weakness of the left leg. 

The patient agreed to a surgical exploration of the lesion for neural decompression and histopathological confirmation. The strong adherence of the mass to neural structures prevented a safe resection, only allowing for a biopsy. A catheter-tip-associated granulomatous mass was then confirmed. Despite the recommendation for the discontinuation of ITMS therapy and substitution with oral opiates, the patient refused due to her history of oral opiate dependency. The severity of the pain did not permit lower daily doses of ITMS. We continued using a 10 mg/ml morphine’s concentration, but this achieved neither a reduction in the size of the CTG nor an improvement of the clinical symptoms.

We suggested a trial of SCS to treat the CTG-associated symptoms as well as the chronic lumbar pain related to FBSS. After a successful trial, the patient underwent implantation of a neuromodulation device in 2018 (Figures 1d, 1e). A paraesthesia-based stimulation program (intensity 13.5 mA, pulse duration 580 µs, and frequency 50 Hz) provided lasting pain relief. Over the course of the next three months, the patient required progressively lower doses of ITMS until complete cessation of intrathecal opiate therapy. A follow-up MRI scan in 2020 revealed a reduction in the size of the granuloma, and the patient has been able to remain without oral opioid analgesics (Figure 1f).

## Discussion

Although initially introduced for cancer pain, IADP has proven to provide satisfactory pain control in other chronic pain conditions for decades [1]. Before electrode-stimulation-based neuromodulation was widely available, IADP was one of the treatments of choice for FBSS patients, who did respond to conventional pharmaceutical and non-pharmaceutical therapies. However, in up to 1% of IADP patients, the formation of an inflammatory granulomatous mass at the tip of the catheter has been reported [1].

Although CTG is most commonly associated with the use of morphine sulfate, other opioid and non-opioid drugs have been also been linked to these lesions [3,4]. Some studies suggest that morphine can act as a mitogen and activate a mitogen-activated protein kinase cascade, and thus lymphocytic activity. It can enhance cytokine formation, leading in turn to an inflammatory cell response, and via nitric oxide release in endothelial cells, granulocytes, and monocytes, it can produce monocyte migration [5].

Treatment of CTG usually involves the discontinuation of drug therapy and surgical removal if neurological symptoms are present [5,6]. There should be a nuanced approach to surgical resection due to the proximity to neurological structures [7]. The management of this rare complication can prove challenging for neurosurgeons and pain specialists. The acute radicular symptoms associated with CTG as well as the exacerbated chronic pain should both be addressed. Removing the granulomatous tissue and replacing intrathecal therapy with oral opiates is not always possible, as demonstrated in this case. A 2019 case report claimed that surgically removing the granuloma without altering the morphine therapy may lead to the recurrence of the lesion [8].

The 2012 Polyanalgesic Consensus Conference (PACC) has recommended lowering the drug concentration and increasing the flow rate in order to prevent progression as well as potentially reduce the size of the granuloma [9]. A 2015 article by Kratsch et al. suggested that higher doses of morphine are associated with the formation of CTG [10]. A Cleveland study on 101 consecutive IADP cases by Veizi et al. showed CTG formation in 8.7% of their cohort, and the authors suggested that low-dose hydromorphone may be associated with an even higher risk of developing this complication [11]. The patient in our study did not experience a reduction in granuloma size despite the lowering of the ITMS concentration. If the continuation of intrathecal drug therapy had been chosen as the treatment plan, long-term low-concentration ITMS therapy or substitution with a different analgesic agent could have eventually reduced the size of the granuloma.

Even non-opiate drugs such as baclofen may cause the formation of catheter-tip-associated inflammatory masses [12]. Kim et al. have reported a case of a patient who had a catheter-tip-associated lesion that was histologically confirmed to be precipitated by morphine after 21 years of intrathecal therapy [13]. The etiopathogenesis of CTG needs further investigation in order to reduce the complication rate of IADP therapy and develop an optimized treatment strategy.

SCS has become a promising therapy option for FBSS [14]; however, its use in acute pain syndromes caused by conditions that cannot be surgically addressed is rarely discussed in the literature. In this case study of an unresectable CTG, the SCS system provided the patient with long-term pain relief and allowed for the discontinuation of all opioid pain medications [13].

## Conclusions

Our patient’s CTG failed to respond to lower concentrations of ITMS as well as surgical resection. The implantation of an SCS device addressed both the chronic FBSS-associated pain as well as the new CTG-associated radicular pain. This allowed for the progressive dilution of the morphine concentration until, eventually, the patient stopped relying on opiate pain medication altogether.
